# Effective Prolonged Therapy with Voriconazole in a Lung Transplant Recipient with Spondylodiscitis Induced by *Scedosporium apiospermum*

**DOI:** 10.1155/2011/460313

**Published:** 2011-08-01

**Authors:** B. Luijk, M. B. Ekkelenkamp, P. A. De Jong, J. M. Kwakkel-van Erp, J. C. Grutters, D. A. van Kessel, E. A. van de Graaf

**Affiliations:** ^1^Division of Heart and Lungs, Departments of Respiratory Medicine and Lung Transplantation, University Medical Center Utrecht, Heidelberglaan 100, 3584CX Utrecht, The Netherlands; ^2^Department of Clinical Microbiology, University Medical Center Utrecht, Utrecht, The Netherlands; ^3^Department of Radiology, University Medical Center Utrecht, Utrecht, The Netherlands; ^4^Department of Pulmonology, St. Antonius Hospital Nieuwegein, Koekoekslaan 1, 3435CM Nieuwegein, The Netherlands

## Abstract

*Scedosporium/Pseudallescheria* species are frequently seen in cystic fibrosis patients. However, disseminated forms after lung transplantation in these patients are rarely seen, but often with poor outcome. In this case report we describe a lung transplant recipient with cystic fibrosis who developed a spondylodiscitis that was caused by *Scedosporium apiospermum*. The patient was treated with anti-fungal treatment by voriconazole for over three years with a clinical good response and without the need for surgical intervention. To our opinion this is the first anti-fungal treated case of invasive disease caused by *Scedosporium/Pseudallescheria* in a cystic fibrosis (CF) patient who underwent lung transplantation that survived.

## 1. Introduction

Fungi are common and serious causes of infections in lung transplant recipients and an incidence of these infections has been reported in 10% to 30%, with a mortality of up to 60% [1]. The most frequently isolated filamentous fungi (moulds) in cystic fibrosis patients are *Aspergillus spp*., followed by* Scedosporium/Pseudallescheria* infections. Invasive infections with *Scedosporium/Pseudallescheria* in CF-patients after lung transplantation are infrequent, but in the current medical literature, invariably fatal [1–3]. Here, we report the first successfully treated case of invasive disease with *Scedosporium/Pseudallescheria* in a cystic fibrosis (CF) patient who underwent lung transplantation. 

## 2. Case

In December 2003, a then 16-year-old female CF patient underwent bilateral lung transplantation. Her CF disease (mutation DF508/1717-1GA) was complicated by exocrine pancreas insufficiency, diabetes mellitus, and decreased growth. Over the years her respiratory tract had been colonized by *Staphylococcus aureus* and *Pseudomonas aeruginosa*, and she suffered from recurrent pulmonary infections with progressive pulmonary dysfunction. Her airways were also colonized by *Scedosporium apiospermum*. In December 2003, she was admitted to the hospital with progression of her chronic respiratory insufficiency, partly due to a severe pulmonary infection with *P. Aeruginosa*. Due to her rapid deterioration she was admitted to the high urgent waiting list for lung transplantation. While awaiting a lung transplant procedure, she received intravenous antibiotic therapy with *ceftazidime* and *tobramycin*, and non-invasive ventilation. In December 2003 she was successfully transplanted, and antibiotics were continued up to 2 weeks after the procedure. Immunosuppressive therapy was instituted with *tacrolimus* and *mycophenolate*, and high first doses of *prednisone* were tapered to a low maintenance dose of 10 mg. Prophylactic antiviral therapy (*valgancyclovir*) was given with cytomegalo virus (CMV) positive donor and recipient. Because of the prior colonization with *Scedosporium apiospermum* prophylactic antifungal therapy was started upon transplantation with voriconazole, which was continued for 6 months according to our hospital protocol that is guided by earlier literature and taking the median time of onset of 4 months into account [2, 4]. Post-transplant her sputum cultures never showed *Scedosporium apiospermum*. Prophylactic antifungal therapy initial regimen after the lung transplantation should be specified. Posttransplant her sputum cultures never showed *Scedosporium apiospermum.* Some more details on postoperative immunosuppressive, antiviral, and antifungal dosage regime would be useful. 

**Figure 1:**
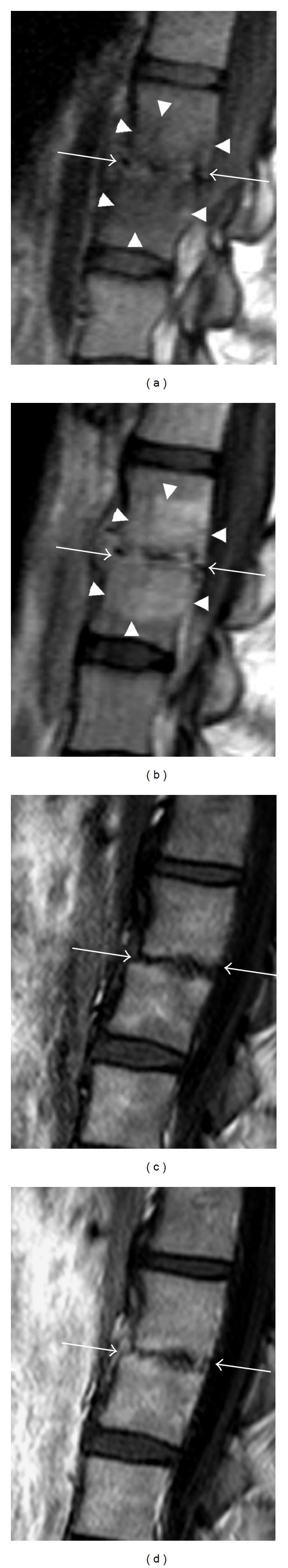
(a) and (b) demonstrates the acute spondylodiscitis with destruction of the intervertebral disk between vertebra lumbar 2 and 3 (arrows). (a) (T1 weighted sagittal MRI scan) demonstrates the edema in the adjacent vertebrae as low signal (arrowheads) and (b) (T1 weighted gadolinium-enhanced sagittal MRI scan) shows the enhancement of the vertebrae as high signal (arrowheads). Imaging findings are consistent with spondylodiscitis with psoas muscle abscess (not shown). After therapy, the bone marrow oedema ((c), T1 weighted sagittal MRI scan) disappeared as did the pathological gadolinium enhancement ((d), T1 weighted gadolinium-enhanced sagittal MRI scan) and the psoas abscess. The destructed disk (arrows) is the only remaining abnormality, consistent with healed spondylodiscitis.

Eight months posttransplantation, in August of 2004, the patient began experiencing lumbar pain without radiation of the pain or fever. On physical examination, there was no tenderness during palpation of lumbar vertebra. Orthopaedic consultancy diagnosed overextension and prescribed physiotherapy and pain medication. Bone scintigraphy showed lumbar microfractures. However, in the following months her lumbar pain did not diminish and therefore a MRI was performed, which demonstrated a spondylodiscitis at the levels L2-L3. She was conservatively treated with a corset and physiotherapy that improved her lumbar pain. In the following 2 years, periodic lumbar X-rays were performed, which did not show increased disruption, and sclerosis was formed. Her lumbar pain disappeared. However, in July 2006, the lumbar pain returned especially during her work and at night. A MRI of the lumbar region was repeated, which showed again a spondylodiscitis. This was interpreted as an active infection of an old focus of spondylodiscitis at L2-L3, with extension of the infection from L1 to L4 (see Figure 1) and abscess formation in the left psoas musculus. A CT-guided fine-needle aspiration of the vertebral lesion showed a negative culture. Therefore, a surgical biopsy was performed. Culture of biopsy was positive for *Scedosporium apiospermum*, proving the fungal aetiology of the infection. Susceptibility testing of the isolate yielded the following minimal inhibitory concentrations (MICs): 0.5 mg/L for *voriconazole*, 2.0 mg/L for *caspofungin*, 0.5 mg/L for *itraconazol*, and 0.25 mg/L for *posaconazol*. 

Antifungal therapy was initiated with *voriconazole* 200 mg twice daily, and the lumbar vertebrae were temporarily immobilised with a corset. The lumbar pain diminished, and after a few weeks, daily activities could slowly be expanded with careful physiotherapy training. A control MRI in July 2007 still showed a spondylodiscitis with disruption of the disci, but the abscess had disappeared. In January 2008 she was treated with *solumedrol* for the suggestion of an acute rejection and in addition, OKT-3, a monoclonal *CD3* antibody (m*uromonab*-*CD3*), was administered. One month later a CMV reactivation occurred for which *valgancyclovir* was given. 

A follow-up MRI (Figure 1) that was performed in December 2009 showed disappearance of active signs of spondylodiscitis. Signs of a destructed disk were the only remaining abnormalities seen on MRI. There were no signs of toxic side effects (liver transaminases were only slightly elevated) after prolonged voriconazole therapy in this patient, and she tolerated it very well. It was decided to discontinue her treatment with *voriconazole* after three and a half years of antifungal therapy. Unfortunately, this year, she again experienced some lumbar pain and a very recent MRI showed signs of a relapse at lumbar level 1 twelve months after discontinuation. We again started *voriconazole* treatment. The effect of this treatment has to be evaluated yet.

## 3. Discussion

*Scedosporium apiospermum* is a filamentous mold present in soil, sewage, and polluted waters [2, 5]. Up to quite recently, it was considered the anamorph (asexual stage) of the mould *Pseudallescheria boydii*, but recent taxonomical studies have revealed that in fact, two different but very closely related species exist [6]. *S. apiospermum* can be distinguished from the anamorph of *P. boydii* through its inability to assimilate D-ribose and the fact that it does not have a sexual stage. The infections caused by the two species are, however, similar, as are their susceptibility patterns and current treatments.

Prior to transplantation, *Scedosporium*/*Pseudallescheria* are isolated from upper or lower airway tract material in 6%–10% of CF patients [3]. Due to the colonization of the sinuses and airways by *Scedosporium,* CF patients are prone for development of an invasive fungal disease after lung transplantation in their concomitant immunosuppressive status [7]. 

Six cases of invasive *Scedosporium*/*Pseudoallescheria* infection in posttransplant CF patients have been described previously [3, 7, 8]. Five cases developed between two and eight weeks after the transplant procedure, one case after seven months. All patients had disseminated disease and/or central nervous system involvement, and, ultimately, none survived the infection. In four patients, colonization was demonstrated prior to the transplant procedure. 

The patient presented in this report is noteworthy, as she had an unusual localization of the infection and because she is the first CF patient reported to survive such an infection. Whether this *Scedosporium apiospermum* infection occurred primarily or secondarily is impossible to determine. When evaluating the onset of infection after lung transplantation, a median time of approximately 4 months has been described, but longer periods have been reported of up to 18 months posttransplantation [2, 4, 8]. However, the long interval between the original complaints and the diagnosis of spondylodiscitis makes it more likely that the disk is secondarily infected. It may be possible that this *Scedosporium* infection developed by haematogenous spread from the colonized sinuses, since sputum cultures did not yield *Scedosporium* after transplant [1]. Possibly, dissemination to other organs did not occur, because she did not require high levels of immunosuppression (i.e., the use of high dose corticosteroids) for acute rejection.

*Voriconazole* is generally the antifungal agent of choice for the treatment of *Scedosporium apiospermum* [1, 7, 8]. However, the interaction of *voriconazole* with calcineurin inhibitors and the elevation of liver enzymes makes adequate dosing in therapeutic levels rather intricate in lung transplant recipients [4], and CF patients may have increased liver metabolism and higher voriconazole clearance [9]. Voriconazole blood levels should, therefore, be controlled during treatment [4]. This was not routinely performed in the past at our department, and we are currently performing voriconazole bloodlevels on a more regular basis in every patient. However, the effect of monitoring *voriconazole* blood levels in lungtransplant recipients on safety issues has still to be evaluated in our clinic.

Other cases suggested that the combination of antibiotic treatment with surgical intervention would give optimal eradication of the infection focus as a good treatment choice in both organ transplant recipients and immunocompetent patients [10–15]. In a recent retrospective analysis of a large number of fungal central nervous system infections (18% *s. apiospermum*), voriconazole showed a good efficacy. Furthermore, prior antifungal therapy to neurosurgery may improve survival [15]. Thus far, we only found one case in the literature that reported successful treatment with *voriconazole* of *Scedosporium Prolificans* without radical surgery in a renal transplant recipient with septic arthritis and probable osteomyelitis [16]. In our case, prolonged *voriconazole* therapy without surgery resulted in clinical recovery of the spondylodiscitis with an excellent improvement on the MRI (see Figure 1). Nevertheless, our patient showed a relapse 12 months after discontinuation of *voriconazole*. Therefore, we suggest that when the *S. apiospermum* infection has become manifest, the antifungal treatment period must be significantly expanded and for some patients to even lifelong therapy [7]. This approach is in agreement with Musk et al., who found that prolonged therapy with *voriconazole* may improve survival and probably by controlling this fungal disease [5]. 

Evidence on the matter remains scarce, but in light of the aggressive course of *Scedosporium* and *Pseudallescheria* infections, it seems prudent to treat all transplant patients colonized with these fungi prophylactically with a minimum of four-to-six months of antifungal therapy and possibly longer when a patient receives high dosages of immunosuppressive agents [4]. Even then, not every case will be prevented, and these microorganisms should always be considered in the differential diagnosis of posttransplant infections.
